# Summary

**Published:** 1853-10

**Authors:** 


*Summary.*—As we are not able to publish entire all valuable articles which appear in other Journals, we have introduced a new department, in which we propose to give a condensed summary of such papers as we cannot transfer without abridgment to our pages, which will be arranged under the following heads: ***Dental Science, Dental Chemistry,*** and ***Progress of Anatomy and Physiology.*** The summary under the second and third heads, will appear in alternate numbers. In this way, we hope to be able to furnish our readers with every thing of interest or value, connected with the progress of dental science, which may from time to time, appear either in this country or Europe. In doing this, we impose upon ourselves a large additional amount of labor; but, we hope to be able to enhance the value of the Journal, so much by it, as to secure a corresponding increase in the number of its subscribers.

In preparing the "quarterly summary," we shall endeavor to give the substance of each article, but shall be compelled, for the most part, to do it in our own language; but where we can, consistently with the brevity necessary to be observed, we shall use the words of the author, placing them, however, in quotation marks, only, when a paragraph is transferred without alteration or abridgment. But we shall endeavor, in all cases, to give his exact meaning, without expressing, in immediate connection, any opinion of our own concerning it.

